# Compatibility: drugs and parenteral nutrition

**DOI:** 10.1590/S1679-45082016AO3440

**Published:** 2016

**Authors:** Talita Muniz Maloni Miranda, Andressa de Abreu Ferraresi

**Affiliations:** 1 Hospital Israelita Albert Einstein, São Paulo, SP, Brazil.

**Keywords:** Drug incompatibility, Parenteral nutrition, Parenteral nutrition, total, Pharmacists, Drug stability

## Abstract

**Objective:**

Standardization and systematization of data to provide quick access to compatibility of leading injectable drugs used in hospitals for parenteral nutrition.

**Methods:**

We selected 55 injectable drugs analyzed individually with two types of parenteral nutrition: 2-in-1 and 3-in-1. The following variables were considered: active ingredient, compatibility of drugs with the parenteral nutrition with or without lipids, and maximum drug concentration after dilution for the drugs compatible with parenteral nutrition. Drugs were classified as compatible, incompatible and untested.

**Results:**

After analysis, relevant information to the product’s compatibility with parental nutrition was summarized in a table.

**Conclusion:**

Systematization of compatibility data provided quick and easy access, and enabled standardizing pharmacists work.

## INTRODUCTION

Calorie *deficit* compromises the provision of energy to our organism, which constitutes a frequent and severe problem at intensive care units. It is also associated with increase of complications, length of hospital stay and mortality.^1^ Parenteral nutrition (PN) is a vital therapeutic modality for newborns, children and young adults under these conditions. Many indications use a variety of electrolytes and nutrients, which is a therapy that might supply calorie *deficit* and, therefore, assure fast and optimal recovery.^2^

The adequate use of this complex therapy provides clinical benefits and reduces potential adverse risks. Complications occur both because of the PN mixture, and also because of process in which it is used.^3^ A review performed in 2004 on safety practices for parenteral nutrition approached the standardization of PN general practices to improve assistance and limits medication errors in this therapy.^4^

Severe patients, in addition to PN, normally need a variety of medications, solutions and blood transfusions.^1^ Co-administration of medications with PN can be need mainly in cases of limited venous access. Therefore, chemical-physical incompatibility can occur between medications and PN.^5^

One of activities of clinical pharmacist is exactly to verify compatibility of injected drugs for infusion in parallel through the same venous access for PN. This verification must be done with extremely careful and constitute one of the last alternatives, because it would, whenever possible, avoid infusion due to risk of chemical-physical incompatibility.

A compatibility study is needed to assure pharmacological conditions for both medications and PN, because this verification can increase safety and avoid formation of incompatible composition during infusion. Incompatibility can occur, for example, due to formation of precipitations that affect negatively and cause delay in recovery of inpatients.^6^

## OBJECTIVE

Standardization and systematization of data to provide quick access to compatibility of leading injectable drugs used in hospitals for parenteral nutrition.

## METHODS

This study was developed by pharmacists of the Department of Clinical Pharmacists of *Hospital Israelita Albert Einstein*, São Paulo (SP), Brazil. Data were collected from July 2014 to March 2015 using the MICROMEDEX^®^ Healthcare Series database from Thomson & Reuters.

We selected 55 injectable drugs commonly used in hospitals. We analyzed compatibility of these medications with PN in the MICROMEDEX^®^ Healthcare Series database from Thomson & Reuters.^7^

This assessment involved two types of PN: 2-in-1 and 3-in-1. The formula 2-in-1 lacks intravenous lipid emulsion; combined glucose and amino acids, and present yellow-like staining because of the presence of multivitamins. The formula 3-in-1 is also known as total nutrient admixture (TNA), it has intravenous lipid emulsion, aminoacids and glucose, and opaque coloration. The presence of lipids in 3-in-1 can change the stability of PN.^8^

We evaluated compatibility of medications with PN 2-in-1 and 3-in-1 considering the final concentration of medications after dilution. The following information were included: active ingredient, compatibility of medications with PN with or without lipids, and maximal concentration of medication after dilution formula of medications compatible with PN. Medications were classified as compatible, incompatible and untested.

## RESULTS

After analysis of each of the 55 selected medications, a table was designed including data of compatibility of medications with PN. Only the compatibility of ranitidine with PN 3-in-1 was not informed because it varies according to composition of PN, therefore it requires the pharmacist’s assessment (Table 1).

**Table 1:** Compatibility of medications with parenteral nutrition 2-in-1 and 3-in-1

Active ingredient	PN without lipids 2-in-1	Maximum concentration	PN with lipids 3-in-1	Maximum concentration	Notes
Aciclovir	INC		INC		
Amikacin	C	250mg/mL	C	5mg/mL	
Aminophylline	C	2.5mg/mL	C	2.5mg/mL	
Ampicillin	C	20mg/mL	C	40mg/mL	
Liposomal amphotericin B	INC		INC		Incompatible with sodium chloride
Caspofungin	INC		NT		
Cefazolin	C	10mg/mL	C	20mg/mL	
Cefepime	C	90mg/mL	C	100mg/mL	
Cefoxitin	C	200mg/mL	C	20mg/mL	
Ceftriaxone	INC		INC		Attention during preparation is required because it includes calcium in composition, which is compatible with ceftriaxone
Ciprofloxacin	INC		C	1mg/mL	Preparation is ready to be used in 2mg/mL concentration
Clarithromycin	NT		NT		
Clindamycin	C	150mg/mL	C	12mg/mL	
Chlorpromazine	C	2mg/mL	C	2mg/mL	
Ciclosporin	C	0.15mg/mL	C	5mg/mL	
Dexamethasone	C	1mg/mL	C	4mg/mL	
Diazepam	NT		NT		
Diphenhydramine	C	50mg/mL	C	50mg/mL	
Dimenhydrinate	NT		NT		
Calcium folinate	C	2mg/mL	C	2mg/mL	
Foscavir	C	24mg/mL	INC		
Furosemide	C	10mg/mL*	C	10mg/mL	PN 2-in-1: compatible only in 10mg/mL concentration*
Ganciclovir	C	1mg/mL	INC		
Hydrocortisone	C	1mg/mL	C	1mg/mL	
Doxycycline	C	10mg/mL	NT		
phenobarbital	C	5mg/mL	INC		
Fentanyl	C	0.05mg/mL	C	0.05mg/mL	
Fluconazole	C	2mg/mL	C	2mg/mL	
Ganciclovir	INC		INC		
Calcium gluconate	C	40mg/mL	C	40mg/mL	
Hydralazine	NT		NT		
Hydroxyzine	C	2mg/mL	NT		
Imipenem/cilastatin	C	10mg/mL*	C	10mg/mL*	Compatible only in 10mg/mL concentration*
Human immunoglobulin	NT		NT		
Regular insulin	C	2UI/mL	C	1UI/mL	
Levofloxacin	NT		NT		
Meropenem	NT		C	50mg/mL	
Mesna	C	10mg/mL	C	10mg/mL	
Methylprednisolone	C	5mg/mL	C	5mg/mL	
Metoclopramide	C	0.58mg/mL	C	5mg/mL	
Metronidazole	C	5mg/mL	C	5mg/mL	
Micafungin	C	1.5mg/mL	NT		
Midazolam	C	0.5mg/mL	INC		
Morphine	C	30mg/mL	C	5mg/mL	
Naloxone	NT		NT		
Oxacillin	C	167mg/mL	C	20mg/mL	
Penicillin G potassium	C	500.000UI/mL	C	40.000UI/mL	
Promethazine	INC		C	2mg/mL	
Ranitidine	C	5mg/mL			Varies according to PN composition, the pharmacist must be contacted to evaluate the formula
Trimethoprim + sulfamethoxazole	C	4mg/mL	C	4mg/mL	
Magnesium sulfate 10%	C	100mg/mL	C	100mg/mL	
Tacrolimus	C	1mg/mL	C	1mg/mL	
Teicoplanin	C	0.2mg/mL	C	0.2mg/mL	
Vancomycin	C	50mg/mL	C	10mg/mL	
Voriconazole	INC		INC		

MICROMEDEX^®^ Healthcare Series. Version 2.0.^(7)^

*compatible only in concentration described in the table, other concentrations can be incompatible or untested. PN: parenteral nutrition; INC: incompatible; C: compatible; NT: untested.

## DISCUSSION

Injectable drugs administration by two separated PN is viable and always preferred. However, several times, simultaneous administration of medications and PN requires considerations regarding compatibility.^1^ According to the decree 272 April 8, 1998, infusion must occur by an single access. When this is not possible, the nutritional therapy team must be contacted in order to provide guidance on possible interaction or other problem that may occur. In the absence of single access route the use of dual pathway infusion pump is common for co-infusion of total parenteral nutrition and other solutions.^9^

There are some alternative to avoid inclusion of medications in the same access route of PN, such as, oral or peripheral intravenous infusion for medication administration, cyclic infusion of PN or the use of multi-lumen catheter.

Medication administration with PN requires knowledge of stability, compatibility and medications interactions with nutritional mixture. There is need of assessment drug-related with pH, diluents, concentration of divalent ions in PN, presence of electrolytes, final concentration of the drug and also the analysis of reports in literature about compatibility.

The summary provided in the table of this study took into consideration information of final concentration of drugs, but there is the need to give special attention to formulation of solutions already in use and which there is no need of drug dilution, such as, the compatible concentration with PN, according with table, which is 1mg/mL. Therefore, the medication is incompatible with PN, because its concentration exceeds the one tested with the PN.

Drugs without compatibilities studies with PN are not recommended to be simultaneous administrated because of the lack of support in the scientific literature for such infusion. For incompatible medications, there is contraindication to be administrated in same PN access for the risk that this incompatibility may pose.

The table of our study has advantages because it standardizes compatibilities information especially for the assessment of possible divergent interpretation that can occur. It also provides clear and concise information, and constitutes a tool for quick and easy access.

A limitation of our study was the need frequent revision of data. Compatibility studies should have data updated as studies are conducted.

## CONCLUSION

The compatibility study of injectable drugs with parenteral nutrition is extremely important because it increases patient safety. Systematization of compatibility data in a table provides easy and quick access, facilitate and standardize the pharmacist’s work.

## References

[1] Robinson CA, Lee JE (2007). Y-site compatibility of medications with parenteral nutrition. J Pediatr Pharmacol Ther.

[2] Calder PC, Jensen GL, Koletzko BV, Singer P, Wanten GJ (2010). Lipid emulsions in parenteral nutrition of Intensive care patients: current thinking and future directions. Intensive Care Med.

[3] Mirtallo JM (2012). Consensus of parenteral nutrition safety issues and recommendations. JPEN J Parenteral Enteral Nutr.

[4] Mirtallo J, Canada T, Johnson D, Kumpf V, Petersen C, Sacks G, Seres D, Guenter P, Task Force for the Revision of Safe Practices for Parenteral Nutrition (2004). Safe practices for parenteral nutrition. JPEN J Parenter Enteral Nutr.

[5] Bouchoud L, Fonzo-Christe C, Klingmüller M, Bonnabry P (2013). Compatibility of intravenous medications with parenteral nutrition: in vitro evaluation. JPEN J Parenteral Enteral Nutr.

[6] Mattox TW, Crill CM, Dipiro JT, Talbert RL, Yee GC, Matzke GR, Wells BG, Posey LM (2011). Parenteral nutrition. Pharmacotherapy: a pathophysiologic approach.

[7] Micromedex (2015). Healthcare Series Database.

[8] Dickerson RN, Sacks GS, Dipiro JT, Talbert RL, Yee GC, Matzke GR, Wells BG, Posey LM (2011). Medication administration considerations with specialized nutrition support. Pharmacotherapy: a pathophysiologic approach.

[9] Brasil, Ministério da Saúde, Secretária de Vigilância Sanitária (2014). Portaria n. 272, de 8 de Abril de 1998. Regulamento técnico para a Terapia de Nutrição Parenteral. Diário Oficial da república Federativa do Brasil.

