# Supplementary material for: Compatibility: drugs and parenteral nutrition
Source: Einstein (Sao Paulo). 2016 Jan-Mar;14(1):52–5. doi: 10.1590/S1679-45082016AO3440 (PMC4872918; doi:10.1590/S1679-45082016AO3440)
Supplement: Translation

# Translation: Compatibilidade: medicamentos e nutrição parenteral

**Authors:** Talita Muniz Maloni Miranda, Andressa de Abreu Ferraresi

**Affiliations:** 1 Hospital Israelita Albert Einstein, São Paulo, SP, Brasil.

**Keywords:** Incompatibilidade de medicamentos, Nutrição parenteral, Nutrição parenteral total, Farmacêuticos, Estabilidade de medicamentos

## Abstract

**Objetivo:**

Padronizar e sistematizar informações, proporcionando um acesso rápido à compatibilidade dos principais medicamentos injetáveis utilizados no âmbito hospitalar para a nutrição parenteral.

**Métodos:**

Foram selecionados 55 medicamentos injetáveis, os quais foram analisados individualmente com dois tipos de nutrição parenteral: dois em um, e três em um. Foram consideradas as seguintes variáveis: princípio ativo, compatibilidade dos medicamentos com a nutrição parenteral com e sem lipídios, e respectiva concentração máxima do medicamento após diluição, para os medicamentos compatíveis com a nutrição parenteral. Os fármacos foram classificados como compatível, incompatíveis e não testado.

**Resultados:**

Após a análise, as informações pertinentes à compatibilidade do medicamento com a nutrição parenteral foram sintetizadas uma tabela.

**Conclusão:**

A sistematização das informações de compatibilidade proporcionou um acesso rápido e fácil, viabilizando e padronizando o trabalho do farmacêutico.

## INTRODUÇÃO

O *déficit* de calorias compromete o fornecimento de energia ao nosso organismo, constituindo um problema comum e grave em unidades de terapia intensiva, e se associando ao aumento de complicações, do tempo de internação e da mortalidade.^1^ A nutrição parenteral (NP) é uma modalidade terapêutica vital para recém-nascidos, crianças e adultos nessas condições. Um grande número de indicações utiliza uma variedade de eletrólitos e nutrientes, terapia esta que intenta suprir o *déficit* de calorias e, assim, garantir uma recuperação rápida e desejada.^2^

O uso adequado dessa terapia complexa proporciona benefícios clínicos e reduz potenciais riscos de eventos adversos. As complicações ocorrem tanto por causa da própria mistura da NP, como por conta dos processos em que é utilizada.^3^ A revisão das práticas de segurança para NP, realizada em 2004, abordou a padronização das práticas gerais de NP para melhorar o atendimento e limitar os erros de medicação envolvendo a terapia.^4^

Para pacientes graves, além da NP, usualmente é necessário o uso de diversos medicamentos, soluções e hemotransfusões.^1^ A administração concomitante de medicamentos com NP pode ser necessária principalmente em casos de limitação de acesso venoso. Desse modo, incompatibilidades físico-químicas podem ocorrer entre os medicamentos e NP.^5^

Uma das atividades do farmacêutico clínico é justamente verificar a compatibilidade dos medicamentos injetáveis para infusão em paralelo, no mesmo acesso venoso da NP. Essa verificação deve ser realizada com extrema cautela e constitui uma das últimas alternativas, pois deve-se, sempre que possível, evitar a infusão, devido a risco de incompatibilidade físico-química.

O estudo de compatibilidade se faz necessário para garantir as condições farmacológicas, tanto dos medicamentos, quanto da NP, já que ele aumenta a segurança e evita à formação de um composto incompatível durante a infusão. A incompatibilidade pode ocorrer, por exemplo, pela formação de precipitados, prejudicando e retardando a recuperação do paciente internado.^6^

## OBJETIVO

Padronizar e sistematizar informações, proporcionando um acesso rápido à compatibilidade dos principais medicamentos injetáveis utilizados no âmbito hospitalar para a nutrição parenteral.

## MÉTODOS

Trata-se de um estudo desenvolvido por farmacêuticos do Departamento de Farmácia Clínica do Hospital Israelita Albert Einstein localizado em São Paulo (SP), utilizando a base de dados MICROMEDEX^®^ Healthcare Series, no período de julho de 2014 a março de 2015.

Foram selecionados 55 medicamentos injetáveis comumente utilizados no ambiente hospitalar. Foi realizada uma análise da compatibilidade destes medicamentos com a NP, utilizando a base de dados MICROMEDEX^®^ Healthcare Series.^7^

Esta avaliação envolveu dois tipos de NP: dois em um e três em um. A fórmula dois em um é isenta de emulsão lipídica intravenosa; combina glicose e aminoácidos; e apresenta uma coloração amarela pela presença de multivitaminas. Já a fórmula três em um, também conhecida como *total nutrient admixture* (TNA) possui emulsão lipídica intravenosa, aminoácidos e glicose, e a coloração é leitosa. A presença de lipídeos na três em um pode mudar a estabilidade da NP.^8^

Foram avaliadas a compatibilidade dos medicamentos com a NP dois em um e três em um, levando em consideração a concentração final do medicamento após diluição. Foram incluídas as seguintes informações: princípio ativo, compatibilidade dos medicamentos com a NP com e sem lipídios, e a respectiva concentração máxima do medicamento após diluição para os medicamentos compatíveis com a NP. Os fármacos foram classificados entre compatível, incompatível e não testado.

## RESULTADOS

Após a análise de cada um dos 55 medicamentos selecionados, foi elaborada uma tabela, que comtemplou as informações pertinentes à compatibilidade do medicamento com a NP. Somente a compatibilidade do medicamento ranitidina com a NP três em um não foi informada, pois variava de acordo com a composição da NP, apresentando recomendação para entrar em contato com o farmacêutico para sua avaliação (Tabela 1).

**Tabela 1:** Compatibilidade dos medicamentos com nutrição parenteral dois em um e três em um

Princípio ativo	NP sem lipídios dois em um	Concentração máxima	NP com lipídios três em um	Concentração máxima	Observação
Aciclovir	INC		INC		
Amicacina	C	250mg/mL	C	5mg/mL	
Aminofilina	C	2,5mg/mL	C	2,5mg/mL	
Ampicilina	C	20mg/mL	C	40mg/mL	
Anfotericina B lipossomal	INC		INC		Incompatível com cloreto de sódio
Caspofungina	INC		NT		
Cefazolina	C	10mg/mL	C	20mg/mL	
Cefepime	C	90mg/mL	C	100mg/mL	
Cefoxitina	C	200mg/mL	C	20mg/mL	
Ceftriaxona	INC		INC		Atenção às preparações que contenham cálcio na composição, incompatível com ceftriaxona
Ciprofloxacino	INC		C	1mg/mL	Preparação pronta para uso na concentração 2mg/mL
Claritromicina	NT		NT		
Clindamicina	C	150mg/mL	C	12mg/mL	
Clorpromazina	C	2mg/mL	C	2mg/mL	
Ciclosporina	C	0,15mg/mL	C	5mg/mL	
Dexametasona	C	1mg/mL	C	4mg/mL	
Diazepam	NT		NT		
Difenidramina	C	50mg/mL	C	50mg/mL	
Dimenidrinato	NT		NT		
Folinato de cálcio	C	2mg/mL	C	2mg/mL	
Foscavir	C	24mg/mL	INC		
Furosemida	C	10mg/mL*	C	10mg/mL	NP dois em um: compatível somente na concentração 10mg/mL*
Ganciclovir	C	1mg/mL	INC		
Hidrocortisona	C	1mg/mL	C	1mg/mL	
Doxiciclina	C	10mg/mL	NT		
Fenobarbital	C	5mg/mL	INC		
Fentalina	C	0,05mg/mL	C	0,05mg/mL	
Fluconazol	C	2mg/mL	C	2mg/mL	
Ganciclovir	INC		INC		
Gluconato de cálcio	C	40mg/mL	C	40mg/mL	
Hidralazina	NT		NT		
Hidroxizine	C	2mg/mL	NT		
Imipenem/cilastatina	C	10mg/mL*	C	10mg/mL*	Compatível somente na concentração de 10mg/mL*
Imunoglobulina humana	NT		NT		
Insulina regular	C	2UI/mL	C	1UI/mL	
Levofloxacino	NT		NT		
Meropenem	NT		C	50mg/mL	
Mesna	C	10mg/mL	C	10mg/mL	
Metilprednisolona	C	5mg/mL	C	5mg/mL	
Metoclopramida	C	0,58mg/mL	C	5mg/mL	
Metronidazol	C	5mg/mL	C	5mg/mL	
Micafungina	C	1,5mg/mL	NT		
Midazolam	C	0,5mg/mL	INC		
Morfina	C	30mg/mL	C	5mg/mL	
Naloxona	NT		NT		
Oxacilina	C	167mg/mL	C	20mg/mL	
Penicilina G potássica	C	500.000UI/mL	C	40.000UI/mL	
Prometazina	INC		C	2mg/mL	
Ranitidina	C	5mg/mL			Varia de acordo com a composição da NP entrar em contato com o farmacêutico para avaliação da formulação
Sulfametoxazol + trimetoprima	C	4mg/mL	C	4mg/mL	
Sulfato de magnésio 10%	C	100mg/mL	C	100mg/mL	
Tacrolimus	C	1mg/mL	C	1mg/mL	
Teicoplamina	C	0,2mg/mL	C	0,2mg/mL	
Vancomicina	C	50mg/mL	C	10mg/mL	
Voriconazol	INC		INC		

MICROMEDEX^®^ Healthcare Series. Versão 2.0.^(7)^

* compatível exclusivamente na concentração descrita na tabela, outras concentrações podem ser incompatível ou não testada. NP: nutrição parenteral; INC: incompatível; C: compatível; NT: não testado.

## DISCUSSÃO

A administração de medicamentos injetáveis em via separada da NP é viável e sempre preferida. No entanto, muitas vezes, a administração simultânea de medicamentos e NP demanda algumas considerações sobre compatibilidade.^1^ De acordo com a portaria 272, de 8 de abril de 1998, a infusão deve ocorrer, em via própria e exclusiva para essa finalidade. Quando isso não for possível, a Comissão de Terapia Nutricional deve ser acionada, para orientar sobre possíveis interações ou outros problemas que possam vir a ocorrer. É comum, na ausência de uma via exclusiva, a utilização de equipos de duas vias, para se infundirem concomitantemente a nutrição parenteral total e outras soluções endovenosas.^9^

Algumas alternativas podem ser sugeridas para evitar a adição de fármacos na mesma via da NP, como, por exemplo, uso da via oral ou intravenosa periférica para administração de medicamentos, correr NP cíclica ou a utilização de cateter multilúmen.

A administração de medicamentos com a NP exige conhecimento da estabilidade, da compatibilidade e das interações do fármaco com a mistura nutricional. Faz-se necessária a avaliação farmacêutica relativa ao pH do medicamento, aos diluentes, à concentração do íons divalentes na NP, à presença de eletrólitos, à concentração final do medicamento e também aos relatos na literatura científica sobre compatibilidade.

A tabela sintetizada neste estudo levou em consideração a informação da concentração final dos medicamentos, mas deve-se ter atenção especial para formulações com soluções já prontas para o uso, para as quais não é necessária a diluição do fármaco, como, por exemplo, o ciprofloxacino, concentração final 2mg/mL. Nesse caso, a concentração compatível com a NP, de acordo com a tabela, é 1mg/mL. Portanto, o medicamento é incompatível com a NP, pois sua concentração excede a testada com a NP.

Para os medicamentos sem estudos de compatibilidade com a NP, não é recomendada a administração simultânea, por não se ter a informação da literatura que embase tal infusão. Para os medicamentos incompatíveis, é contraindicada a administração na mesma via da NP, pelo risco que a incompatibilidade oferece.

Essa tabela tem a vantagem de padronizar as informações de compatibilidade, pois, durante a avaliação, interpretações divergentes podem ocorrer. A tabela disponibiliza as informações de forma clara e objetiva, além de ser uma ferramenta de busca rápida, que pode ser consultada a qualquer momento.

Uma limitação deste estudo foi o fato de as informações disponíveis necessitarem ser revisadas com períodos regulares. Estudos de compatibilidade devem ter as informações atualizadas conforme os estudos forem realizados.

## CONCLUSÃO

O estudo de compatibilidade dos medicamentos injetáveis com a nutrição parenteral é de extrema importância para aumentar a segurança do paciente. A sistematização das informações de compatibilidade em forma de tabela proporcionou um acesso rápido e fácil, viabilizando e padronizando o trabalho do farmacêutico.
